# Bistable Expression of Virulence Genes in *Salmonella* Leads to the Formation of an Antibiotic-Tolerant Subpopulation

**DOI:** 10.1371/journal.pbio.1001928

**Published:** 2014-08-19

**Authors:** Markus Arnoldini, Ima Avalos Vizcarra, Rafael Peña-Miller, Nicolas Stocker, Médéric Diard, Viola Vogel, Robert E. Beardmore, Wolf-Dietrich Hardt, Martin Ackermann

**Affiliations:** 1 Institute of Biogeochemistry and Pollutant Dynamics, Department of Environmental Systems Science, ETH Zurich, Zurich, Switzerland; 2 Department of Environmental Microbiology, Eawag, Dübendorf, Switzerland; 3 Laboratory of Applied Mechanobiology, Department of Health Science and Technology, ETH Zurich, Zurich, Switzerland; 4 Centro de Ciencias Genómicas, Universidad Nacional Autónoma de México, Mexico City, Mexico; 5 Institute of Microbiology, Department of Biology, ETH Zurich, Zurich, Switzerland; 6 Biosciences, University of Exeter, Exeter, United Kingdom; Hebrew University, Israel

## Abstract

Phenotypic heterogeneity can confer clonal groups of organisms with new functionality. A paradigmatic example is the bistable expression of virulence genes in *Salmonella typhimurium*, which leads to phenotypically virulent and phenotypically avirulent subpopulations. The two subpopulations have been shown to divide labor during *S. typhimurium* infections. Here, we show that heterogeneous virulence gene expression in this organism also promotes survival against exposure to antibiotics through a bet-hedging mechanism. Using microfluidic devices in combination with fluorescence time-lapse microscopy and quantitative image analysis, we analyzed the expression of virulence genes at the single cell level and related it to survival when exposed to antibiotics. We found that, across different types of antibiotics and under concentrations that are clinically relevant, the subpopulation of bacterial cells that express virulence genes shows increased survival after exposure to antibiotics. Intriguingly, there is an interplay between the two consequences of phenotypic heterogeneity. The bet-hedging effect that arises through heterogeneity in virulence gene expression can protect clonal populations against avirulent mutants that exploit and subvert the division of labor within these populations. We conclude that bet-hedging and the division of labor can arise through variation in a single trait and interact with each other. This reveals a new degree of functional complexity of phenotypic heterogeneity. In addition, our results suggest a general principle of how pathogens can evade antibiotics: Expression of virulence factors often entails metabolic costs and the resulting growth retardation could generally increase tolerance against antibiotics and thus compromise treatment.

## Abstract 2

The bistable expression of virulence genes in *Salmonella* allows a clonal population to hedge its bets: one subpopulation suffers a growth cost, but is tolerant to antibiotics.

## Author Summary

Scientists have recently realized that nature and nurture are not the only determinants of an individual's traits; some organisms also use random molecular processes to generate phenotypic variation among genetically identical individuals. This raises the question of whether such phenotypic variation could be beneficial and what such possible benefits might be. Working with pathogenic *Salmonella* bacteria, we discovered that phenotypic variation in one single trait—the expression of virulence genes—provides this pathogen with two critical benefits. First, it leads to the division of labor between different phenotypic variants that allows for effective host colonization, and second, it provides tolerance to antibiotics through a “bet-hedging” mechanism. Our results provide a new perspective on how phenotypic differences between individuals can provide benefits to clonal groups of organisms. At the same time, this study contributes to explaining why some pathogens can evade treatment, and could help to find new and better ways for controlling infectious disease.

## Introduction

Genetically identical bacterial cells can exhibit remarkable phenotypic differences even when grown in homogeneous environments [1],[2]. These differences can arise from stochastic fluctuations in the expression of individual genes [3]. Although there is evidence that the majority of genes are under selection for tight control of expression [4], some genes are expressed heterogeneously. This raises the question of whether phenotypic heterogeneity can provide benefits and what those benefits might be.

Two possible types of benefits have been proposed. First, heterogeneous gene expression can enable a population to hedge its bets in an unpredictable and fluctuating environment [3],[5]–[7]. In this bet-hedging scenario, one part of the population expresses a phenotype optimized for the current environment, allowing it to survive and reproduce at a high rate. Another part of the population expresses a phenotype less well suited to the current environment, yet it is adapted to a state the environment might change into. Second, phenotypic heterogeneity can promote the division of labor in groups of genetically identical individuals [8]–[10]. This allows a population to perform different functions simultaneously that would be costly or impossible to combine within a single individual. Bet-hedging and division of labor are two fundamentally different adaptive strategies: The benefit of bet-hedging only manifests in fluctuating environments over time; the benefit of division of labor does not require environmental fluctuations to manifest, and the payoff to each subpopulation depends on the interaction with the other subpopulation. Both strategies have been shown independently to play important roles in microbial populations [8]–[13]. Whether heterogeneity in a single trait can promote both functions simultaneously, and how these functions can interact, is an open question. Our aim is to address this question and thereby to gain new insights into the functional complexity of phenotypic heterogeneity.

Here, we present a case where phenotypic heterogeneity in a single trait—virulence gene expression in *Salmonella typhimurium*—shows characteristics of both strategies, the division of labor and bet-hedging. In *S. typhimurium*, expression of the type three secretion system 1 (*ttss-1*) is bistable [14]–[16]. *S. typhimurium* uses *ttss-1* for injecting effector proteins into host cells, promoting penetration of the host tissue. It is, therefore, an important determinant of virulence in this pathogen [17]. It has been shown that the bistable expression of *ttss-1* leads to the division of labor among the members of a population. One subpopulation expresses *ttss-1* (T1^+^ cells) and a fraction of those cells invade host tissue and evoke an inflammatory response that is beneficial for the *S. typhimurium* cells that do not invade [18],[19]. This is thus a special case of “cooperative virulence” where the cooperative behavior is only expressed by a fraction of the population. Recently, it has also been shown that members of the T1^+^ subpopulation have low cellular growth rates [9],[20]. Slow growth has been associated with tolerance to environmental stresses such as exposure to antibiotics [11],[21]–[25], and the formation of a slow-growing and persistent subpopulation has been interpreted as a typical example for bet-hedging in other organisms [11],[26]. This raised the question of whether the slowly growing T1^+^ subpopulation is more tolerant to antibiotic exposure than the faster growing T1^−^ subpopulation, so that the formation of these two subpopulations could promote bet-hedging during exposure to antibiotics. Although this question does not imply that exposure to antibiotics was the selective force that might have promoted phenotypic heterogeneity in virulence gene expression, it is interesting to ask whether a bet-hedging benefit under exposure to antibiotics is a potentially very relevant consequence of this heterogeneity.

## Results and Discussion

**Figure 1:**
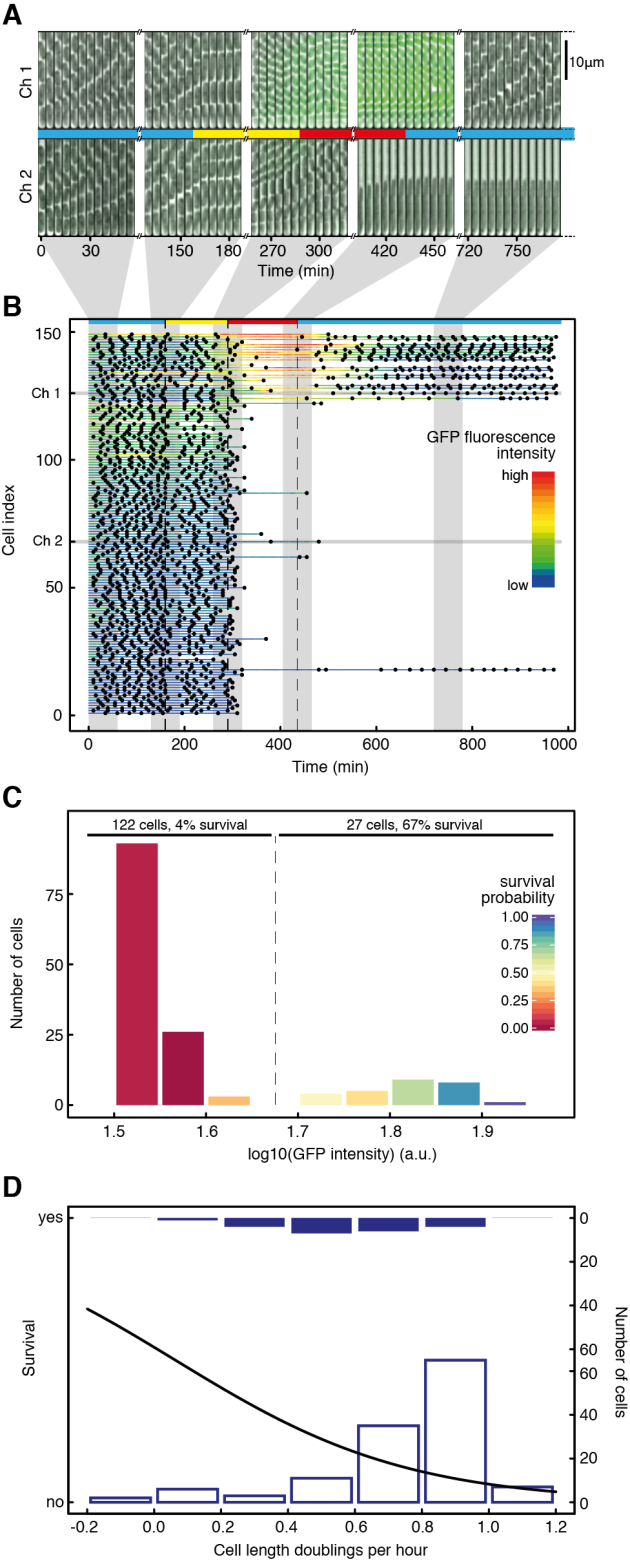
Expression of *ttss-1* is associated with tolerance to antibiotic exposure. (A and B) We used time-lapse analysis of single cells to study tolerance to antibiotics. Bacteria were first grown in LB medium (marked blue), shifted to spent LB at 160 min (yellow), shifted to spent LB containing 0.05 µg/ml ciprofloxacin at 290 min (red), and shifted back to LB at 435 min (blue). (A) Bacterial cells were grown in microfluidic devices in dead-ended channels, with medium flowing through a bigger main channel orthogonal to them. The top row shows a temporal montage of images of a channel in which the bottom cell started expressing *ttss-1* and resumed division after exposure to 0.05 µg/ml ciprofloxacin (“Ch 1”), and the bottom row shows a temporal montage of images of a channel in which the bottom cell did not express *ttss-1* and did not resume division after exposure to antibiotic (“Ch 2”). For each channel, five sets of still images from different phases of the experiment are shown. Each set consists of 12 images recorded at 5-min time intervals. (B) Quantitative analysis of all 149 cells from this experiment. Every horizontal line represents data for an individual cell over 975 min. Dots mark the time points at which this cell divided. Cells are sorted according to *ttss-1* expression during antibiotic exposure, measured as mean GFP fluorescence during that time interval. The cell index indicates the rank of a cell according to its mean GFP expression during antibiotic exposure; a lower cell index indicates lower GFP expression, and a higher cell index indicates higher GFP expression. The color of the lines indicates real-time GFP intensity. Shading indicates the data corresponding to the cells shown in (A). Three independent experiments were performed, all of them showing significant positive correlations of survival with *ttss-1* expression (logistic regression with ANOVA, *p* = 8.8×10^−14^, 2.4×10^−5^, 1.3×10^−8^; *N* = 149, 137, and 144), indicating that T1^+^ cells preferentially survive antibiotic exposure. (C) Histogram of cells in different GFP categories. Color-coding of the columns denotes the probabilities to survive exposure to 0.05 µg/ml ciprofloxacin. The columns were assigned visually to two categories according to GFP intensity (“GFP on,” “GFP off”), and the percentage of cells surviving in the different categories was calculated. (D) Analysis of the single cell elongation rates 75 min before and 25 min after addition of antibiotic and survival. The black curve indicates the survival probability depending on the cell elongation rate as determined by a logistic regression model. The histograms show how many cells were in the respective ranges of elongation rates, and whether they survive antibiotic treatment (full bars, top) or die (empty bars, bottom). For all three experiments, survival was negatively correlated with single cell elongation rates (logistic regression with ANOVA, *p* = 7.8×10^−4^, 3.2×10^−4^, 1.0×10^−4^, *N* = 151, 137, and 114).

To investigate whether the two subpopulations—T1^+^ and T1^−^—exhibit a difference in susceptibility to antibiotics, we grew clonal populations of *S. typhimurium* in a microfluidic device that allows single cell observation over extended periods of time under precisely controlled conditions, and quantifying cellular parameters of large numbers of individual cells [27] (Figure 1A shows a temporal montage of two microfluidic channels, and Figure S1 shows a schematic drawing of the microfluidic device). During exponential growth in LB medium, the majority of cells are T1^−^, which is consistent with previous results [20]. We use filtered medium from late exponential phase cultures grown in LB (“spent LB”) to induce the expression of *ttss-1* [20], which, by virtue of its bistable expression, leads to the emergence of two phenotypic subpopulations. The first subpopulation remains T1^−^, whereas the second induces the expression of *ttss-1* (as observed based on a reporter for *sicA* promoter activity; the *sicA* promoter controls expression of the *sicAsipBCDA* operon, encoding key parts of the *ttss-1* virulence system). Importantly, and as we will show in more detail below, the T1^+^ subpopulation pays a cost for the expression of *ttss-1*, and grows and divides at a slower rate, in line with previously published observations [20]. To test for differential susceptibility of the two subpopulations, we then added 0.05 µg/ml ciprofloxacin. After 3 h of ciprofloxacin exposure, the medium was changed to fresh LB to wash out the antibiotic and to allow growth of surviving cells. Survival of ciprofloxacin treatment showed a positive correlation with single cell GFP intensities (Figure 1B and Figure 1C) and a negative correlation with single cell elongation rates (Figure 1D), and thus with the expression of *ttss-1* (Movie S1); T1^+^ cells, having a growth deficit in the absence of antibiotics, were more likely to survive ciprofloxacin exposure than T1^−^ cells. The bistable expression of *ttss-1* can therefore have two functional consequences: In addition to promoting division of labor between two phenotypes, it can also promote persistence of the genotype in the face of fluctuating exposure to antibiotics through a bet-hedging mechanism.

We then carried out two important control experiments. First, we subjected a strain that is genetically avirulent (Δ*hilD*) to the same experimental conditions as used in Figure 1. HilD is a positive regulator of *ttss-1*, and deletion of *hilD* yields a population of fast growing T1^−^ individuals [20]. None of the observed Δ*hilD* cells resumed division after exposure to 0.05 µg/ml ciprofloxacin (Figure S2), showing that the function of HilD is required to survive antibiotic exposure. Second, we tested whether the tolerance observed in the T1^+^ subpopulation is due to the acquisition of genetic resistance. We subjected cells that had survived exposure to 0.05 µg/ml ciprofloxacin to treatment with the same antibiotic a second time, without inducing expression of *ttss-1* through growth in spent LB. None of the observed cells resumed division after the second antibiotic exposure (Figure S3, Movie S2), indicating that antibiotic tolerance is a phenotypic trait, rather than the result of a resistance mutation.

**Figure 2:**
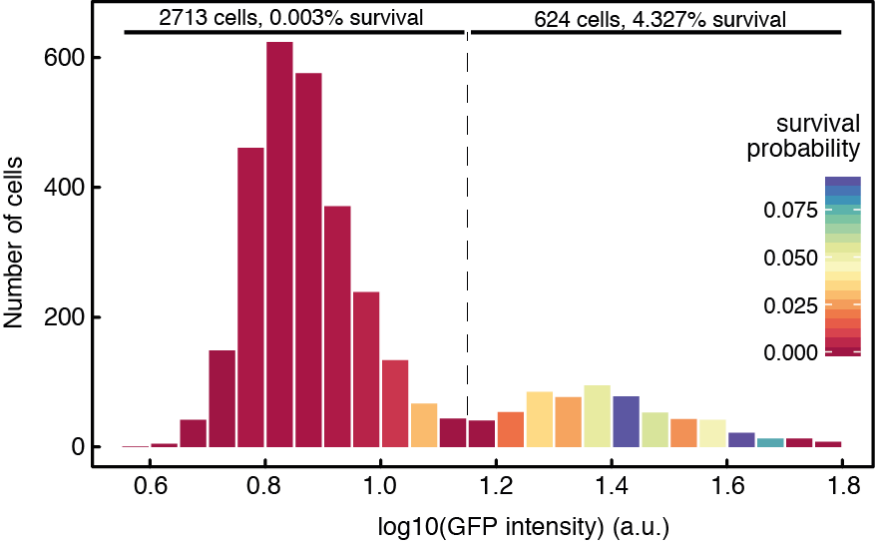
Tolerance of T1^**+**^ cells is also observed at a clinically relevant antibiotic concentration. Pooled results of three independent experiments analogous to the one shown in Figure 1, except that cells were exposed to a higher ciprofloxacin concentration, 10 µg/ml. We determined the *ttss-1* expression levels of a total of 3,337 cells (measured as GFP fluorescence intensity at the last time point during antibiotic exposure) and recorded their fate during exposure to antibiotics. The histogram shows the number of cells in different GFP intensity categories, indicating *ttss-1* expression levels. Background fluorescence intensity (measured in areas of the image not containing cells) was subtracted from the measured GFP values in order to allow pooling of different experiments. Color-coding denotes the probabilities to survive exposure to 10 µg/ml ciprofloxacin for each GFP intensity category. In three independent experiments, cells that express *ttss-1* have a significantly higher survival probability (logistic regression with ANOVA, *p* = 0.03, 6.2×10^−8^, 4.2×10^−3^; *N* = 653, 1,208, and 1,476). In addition, columns were assigned visually to two categories according to their GFP expression (“GFP on,” “GFP off”), and the percentage of cells surviving in the different categories was calculated.

Next, we tested if our results were specific to antibiotic class and concentration. To test whether the differential killing of the two subpopulations is also observed with an antibiotic from a different class, we treated *S. typhimurium* cells with 16 µg/ml kanamycin, otherwise using the same experimental conditions as in Figure 1. Kanamycin is an aminoglycoside and has a fundamentally different mechanism of action from ciprofloxacin, a fluoroquinolone. Again, survival of antibiotic treatment was positively correlated with GFP fluorescence and negatively correlated with single cell elongation rates, and thus to expression of *ttss-1* (Figure S4, Movie S3). Second, we tested whether the tolerance of T1^+^ cells plays a role at antibiotic concentrations that are in the range of those measured in patients treated with ciprofloxacin [28] and kanamycin [29], respectively. Using the same experimental setup as above, we subjected *S. typhimurium* to a ciprofloxacin concentration of 10 µg/ml and a kanamycin concentration of 50 µg/ml. These concentrations are higher than the ones we have used before, and correspond to 200 times the minimal inhibitory concentration (MIC) measured for ciprofloxacin, and 6.25 times the MIC measured for kanamycin (Figure S5). Although more cells died in total when subjected to those higher antibiotic concentrations, survival of T1^+^ cells was again significantly higher compared to T1^−^ cells for both antibiotics tested (Figure 2 and Movie S4 for ciprofloxacin treatment, and Figure S6 for kanamycin treatment). This partial tolerance of T1^+^ cells against clinically relevant concentrations of antibiotics could potentially explain the observation of relapsing *S. typhimurium* infections after treatment [30].

Our results raise the question of whether the growth difference between the T1^+^ and T1^−^ subpopulations can explain the difference in survival. We tested this in two different ways. First, we grew genetically avirulent Δ*hilD* cells in chemostats at the two different growth rates observed for the T1^−^ and the T1^+^ subpopulations, respectively. These growth rates were determined in an experiment where wild-type *S. typhimurium* cells were grown for an extended period of time in spent LB, and single cell growth rates of T1^−^ and T1^+^ cells were determined to be 0.96 and 0.26 doublings per hour, respectively (see Materials and Methods for details). The Δ*hilD* strain allowed us to test the effect of growth rate in a phenotypically uniform population. When treated with 0.05 µg/ml ciprofloxacin, viability counts in the chemostat populations remained largely stable for the slow growth condition (corresponding to the rate at which the T1^+^ subpopulation grows, 0.26 doublings per hour) over a period of 5 h, whereas viability counts for the fast growth condition (corresponding to the rate at which the T1^−^ subpopulation grows, 0.96 doublings per hour) dropped sharply during the first 3 h and then remained stable at around 1 in 10^5^ cells of the initial population (Figure S7). As a second way to reduce cell growth, we manipulated Δ*hilD* cells into overexpressing LacZ, a gratuitous protein under the growth conditions used, using an IPTG-inducible promoter on a high copy plasmid [31]. Again, we observed a strong negative correlation between single cell growth rate and survival (Figure S8, Movie S5). Cells lacking the plasmid do not survive when exposed to the same IPTG concentrations (unpublished data). We therefore conclude that the growth rate difference between the T1^+^ and T1^−^ subpopulations can explain a substantial part of the difference in antibiotic susceptibility, and that the expression of abundant protein upon *ttss-1* induction is a plausible reason for this growth deficiency.

**Figure 3:**
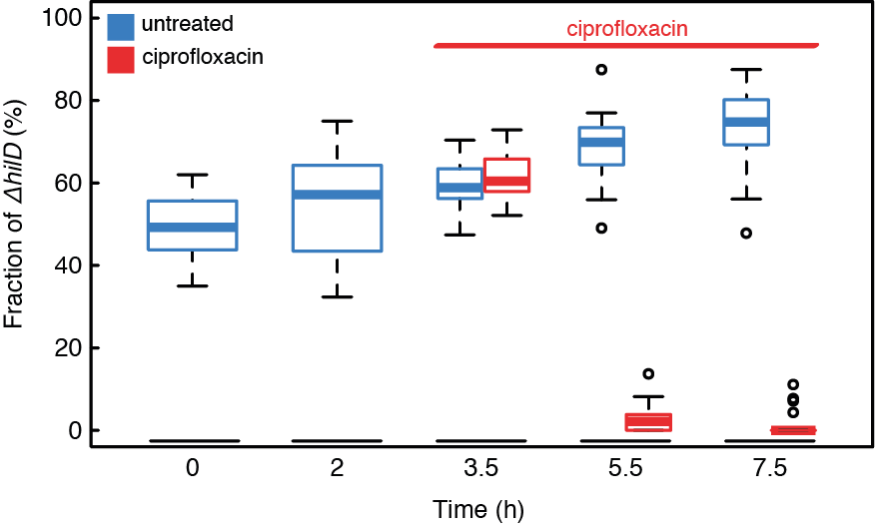
Selection for genetically avirulent mutants is reversed when exposed to antibiotic. Dynamics of the fraction of *ΔhilD* cells in mixed cultures with wild-type cells over time. Cultures were inoculated with approximately 1∶1 ratios of the two strains. A kanamycin resistance marker was used to distinguish between strains. Without antibiotic challenge, *ΔhilD* cells increase in frequency relative to wild-type cells (blue boxes), whereas exposure to 0.05 µg/ml ciprofloxacin reverses the trend, and *ΔhilD* cells decrease in frequency relative to wild type (red boxes). Boxes span the range between upper and lower quartile; thick lines denote the median; whiskers denote the highest and lowest values still within 1.5 interquartile ranges of the upper and lower quartiles, respectively; empty circles represent data points that are outside this range. The addition of ciprofloxacin has a significant influence on the outcome of competition (two way ANOVA, Time×Treatment interaction, *p*<2×10^−16^, *N* = 20).

The link between virulence gene expression and tolerance against antibiotics that we observe has potential consequences for within-host evolution of virulence. In experimental model systems of cooperative virulence [9],[32]–[34] as well as in a clinical setting [35] it has been shown that genetically avirulent mutants can rise in frequency during infection, leading to improved host condition. If exposure to antibiotics kills phenotypically avirulent cells preferentially, one would expect selection against the emergence of such genetically avirulent mutants. In order to test this, we competed the genetically avirulent *S. typhimurium* mutant Δ*hilD* against wild-type *S. typhimurium* cells in the presence and absence of ciprofloxacin *in vitro* (Figure 3). In the absence of antibiotic, we saw an increase in prevalence of the genetically avirulent Δ*hilD* strain, as reported previously [20]. If antibiotic was added to the culture, overall population viability counts dropped (Figure S9) and we observed the opposite effect: The wild-type increased relative to the avirulent Δ*hilD* mutant (Figure 3). The observation that exposure to antibiotics can lead to selection against genetically avirulent mutants *in vitro* raises the question of whether antibiotic treatment could contribute to the maintenance of virulence in a clinical context. These findings also suggest that the two established functions of phenotypic heterogeneity—division of labor and bet-hedging—can interact. Variation in virulence expression in clonal populations can translate into differential susceptibility to antibiotics and lead to a bet-hedging benefit, which could in turn protect clonal populations against the invasion of avirulent mutants [9],[32]–[35] that exploit and subvert the division of labor within these populations [8],[9]. This reveals a new level of complexity in the functional consequences of phenotpyic heterogeneity.

### Conclusions

In this study, we observed a connection between virulence gene expression and tolerance to antibiotics that could be general: The expression of virulence factors often entails metabolic costs [20],[35],[36], possibly as a side effect of the expression of abundant proteins, and the resulting growth retardation could generally increase tolerance against antibiotics and thus compromise treatment. Under this scenario, pathogens would show tolerance to antibiotics even in situations where treatment was not an important selective factor in their evolutionary past. Understanding the cellular basis of antibiotic tolerance, and the consequences it can have on selection for virulence, is important for using existing treatment options effectively and for developing new strategies for controlling pathogens. In addition, our results suggest a general mechanism that could contribute to the evolutionary stability of cooperative behavior in microorganisms: If individuals that express a costly cooperative trait are also better protected against environmental impacts, then this could lead to a stabilization of the cooperative phenotype.

## Materials and Methods

### Media and Chemicals

LB Lennox (Sigma) was used as the growth medium for preculturing for all experiments and as the growth medium in microscopy experiments where indicated. Ampicillin (AppliChem) was added at 100 µg/ml to the growth medium when required. LB buffered with mineral salts [37] was used for growth in chemostats. For the medium used in microscopy experiments, BSA (Sigma) and salmon sperm DNA (Sigma) was added to the medium at 150 µg/ml and 50 µg/ml, respectively, to avoid sticking of the cells to PDMS. Spent medium was obtained by growing the same strain as used in the respective experiments in LB without antibiotics, and by filter sterilizing it when the culture reached an OD 600 nm of 0.8–0.9. Ciprofloxacin (Fluka) was used at a concentration of 0.05 µg/ml for the experiments shown in Figure 1, Figure 3, Figure S2, Figure S3, Figure S7, Figure S8, and Figure S9, and at a concentration of 10 µg/ml for the experiments shown in Figure 2. Kanamycin (Sigma) was used at a concentration of 16 µg/ml for the experiment shown in Figure S4 and at a concentration of 50 µg/ml for the experiment shown in Figure S6. Plates containing 50 µg/ml kanamycin were used to determine strain ratios and colony forming units in the experiment shown in Figure 3 and Figure S9. For the experiment shown in Figure S8, spent LB was supplemented with IPTG (Promega) at the indicated concentrations.

### Strains and Growth Conditions

All strains are derivatives of *S. typhimurium* SL1344 [38] (see Table S1 for a list of all strains used). Bacteria were grown overnight in culture tubes (100 mm×16 mm PP reaction tube, Sarstedt, Nümbrecht, Germany) in 5 ml LB shaking at 220 rpm at 37°C, and then diluted 1∶100 in LB 2–3 h before the experiments to obtain exponentially growing cells in steady state. MICs for ciprofloxacin and kanamycin were determined by the standard method ([39], and Figure S5), and a concentration of 2× MIC (Figure S5 and Text S1) was used for antibiotic treatment in all experiments except for the experiments shown in Figure 2 and Figure S6. For microscopy experiments, the flagella mutant strain X8602 [40] was used to avoid loss of cells from the channels. The plasmid *psicA gfp* [20] driving expression of gfpmut2 from the *sicA* promoter was introduced in all strains used for microscopy and flow cytometry, and GFP expression from this plasmid was used to assess induction of *ttss-1*. For the experiment in Figure S2, a *hilD* deletion allele from strain M2007 was P22 transduced into the X8602 background to yield strain M3139, and subsequently transformed with the *psicA gfp* plasmid. For the experiment in Figure 3 and Figure S9, 10 clones (cultures grown from single colonies) of a kanamycin-sensitive wild-type strain (SB300) were competed against 10 clones of a kanamycin-resistant *ΔhilD* strain (M2007) [20], and 10 clones of a kanamycin-resistant wild-type (resistance cassette inserted at the *lpfED* locus, showing an identical *ttss-1* expression pattern to the kanamycin-sensitive wild type; Figure S9) were competed against 10 clones of a kanamycin-sensitive *ΔhilD* strain (Z19). Deletion mutants were constructed via lambda red recombination as described in [41] and P22 transduced into the clean SB300 or X8602 background, respectively. For the experiment shown in Figure S8, the plasmid pCA24N-lacZ from the ASKA(–) collection [31] was transformed into M3139.

### Microfluidics

The microfluidic devices were made using a design adapted from the one published by Wang et al. [27] (Figure S1). Masks for photolithography were ordered at ML&C GmbH, Jena, Germany. Two-step photolithography was used to obtain silicone wafers. PDMS (Sylgard 184 Silicone Elastomer Kit, Dow Corning) was mixed in a ratio of 10∶1, mixed by stirring, poured on the dust-free wafer, degassed in a desiccator until no visible air bubbles were present, and incubated overnight at 80°C for curing. PDMS chips of approximately 1.5 cm×3.5 cm were cut out around the structures on the wafer. Holes for medium supply and outlet were punched using 18G needles (1.2 mm×40 mm) that were modified by breaking off the beveled tip and sharpening the edges of the then straight tip. Chemical activation of surface residues on the PDMS chips and on 24 mm×40 mm glass coverslips (Menzel-Gläser, Braunschweig, Germany) was performed by treating them for 6 min in a UV-Ozone cleaner (Novascan PSD-UV). The PDMS chips were then placed on the glass coverslips, the exposed sides facing each other, and put on a heated plate at 90°C overnight for binding. Before an experiment, chips were rinsed with LB containing BSA and salmon sperm DNA (concentrations as mentioned above, 2 ml/h pump speed) until the growth channels were filled. Cells from an early exponential phase culture were concentrated approximately 100× by centrifugation (12,470× *g*, 2 min) and loaded into the chip using a pipette. The process of cells entering the channels was observed microscopically, and when sufficient occupation of the channels was observed (after 10–20 min), medium was pumped through. For all experiments, syringe pumps (NE-300, NewEra Pump Systems) with 60 ml syringes (IMI, Montegrotto Terme, Italy) containing the media were used. Tubing (Microbore Tygon S54HL, ID 0.76 mm, OD 2.29 mm, Fisher Scientific) was connected to the syringes using 20G needles (0.9 mm×70 mm), which were directly inserted into the tubing. Smaller tubing (Teflon, ID 0.3 mm, OD 0.76 mm, Fisher Scientific) was then inserted into the bigger tubing and directly connected to the inlet hole in the PDMS chip. Medium change was performed by disconnecting the bigger and smaller tubings and reconnecting to the bigger tubing of a second medium supply. All experiments were run at a pump speed of 2 ml/h.

### Microscopy

Microscopy was performed using an Olympus IX81 inverted microscope system with automated stage, shutters, and a laser-based ZDC autofocus system. Several different positions were monitored in parallel on the same device, and phase contrast and fluorescence images (where applicable) of every position were taken every 5 min. Images were acquired using an UPLFLN100xO2PH/1.3 phase contrast oil immersion objective (Olympus) and a cooled CCD camera (Olympus XM10). For image acquisition, the CellM software package (Olympus) was used. Fluorescence images were acquired using a 120W mercury short arc lamp (Xcite 120PC Q) and the U-N41001 GFP filter set (450–490 nm ex/500–550 em/495 dichroic mirror, Chroma). The whole microscope was placed in an incubated box (Life Imaging Services, Reinach, Switzerland) at 37°C during all experiments.

### Image Analysis

Images were analyzed using the plugin MMJ (available at https://github.com/penamiller/mmJ) for ImageJ [42]. It allows extracting of fluorescence intensities and cell length for the bottom cell of each channel during the course of the whole experiment, and scores division events based on cell length. For analysis of the experiments shown in Figure 1, Figure S2, Figure S3, Figure S4, Figure S8, and for determining growth rates, the standard version of MMJ was used. For the experiments shown in Figure 2 and Figure S6, a modified version of MMJ (MMJAll) was used that allows the extraction of all cells on single frames. Data were then further processed and plots were generated using R [43]. Cells were counted as surviving if they divided at least once after removal of antibiotic.

### Competition

For competition experiments, strains were mixed in a 1∶1 ratio in fresh LB, according to their optical density in overnight cultures. After 3.5 h of growth, all cultures were diluted 1∶100 in spent LB to extend the time cells spend in an induced state, and 0.05 µg/ml ciprofloxacin was added where indicated. Samples were taken at the indicated times, optical density was measured, and dilutions were spread on LB agar plates. After overnight growth, colonies were counted on the LB agar plates, and replica plated on LB agar plates containing 50 µg/ml kanamycin. Surviving colonies on LB kanamycin plates were counted the next day, and ratios of strains were determined. To control for a possible influence of the placement of the kanamycin resistance marker, the experiment was performed with two different strain combinations, 10 replicates each: Kanamycin-sensitive wild type (SB300) was competed against kanamycin-resistant *ΔhilD* (M2007) and kanamycin-resistant wild type (resistance cassette inserted at the *lpfED* locus) were competed against kanamycin-sensitive *ΔhilD* (Z19). Statistical analysis showed no significant influence of maker placement on the time-dependent relative frequency of the strains (three way ANOVA, treatment×time×marker *p* = 0.19). Data from both strain combinations were pooled for the plots shown in Figure 3 and Figure S9.

### Determination of Single-Cell Growth Rates

To determine the growth rates of the T1^+^ and T1^−^ subpopulations, we grew *ΔfliCΔfljB psicA gfp* cells in the same microfluidic devices as used in Figure 1. After initial growth in LB for 2 h, 45 min, we changed the medium to spent LB (see above), which still contains enough nutrients to sustain growth, and monitored growth and gene expression for 13 h, 45 min. We identified all cells (11 cells in total) in the experiment whose fluorescence levels were higher than 10 standard deviations above the fluctuations in background fluorescence (i.e., fluorescence of areas not containing cells in the vicinity of the respective cells measured), and determined the number of cell divisions during that time. To determine the growth rate of T1^−^ cells, we used 11 cells from channels neighboring channels harboring T1^+^ cells that do not show a significant increase in fluorescence and determined the number of doublings of those cells during the same time period as for the T1^+^ cells in the neighboring channel.

### Determination of Single Cell Elongation Rates in Microscopy Experiments

Data on cell length and divisions were extracted for a period of 100 min (20 frames)—75 min (15 frames) before and 25 min (5 frames) after addition of the antibiotic—and linear regression was performed on the natural logarithm of cell lengths between divisions, between the start of the period and the first division in the period, and between the last division in the period and the end of the period, respectively, to determine the slope of the length increase during every division. Arithmetic means of the slopes of every individual cell were then calculated and multiplied by 12 to get a number for length doublings per hour.

### Chemostats

Chemostat growth was performed using a Sixfors system (Infors HT, Bottmingen, Switzerland) with six parallel reactors. Buffered LB was used as growth medium, as described in Ihssen et al. [37]. We inoculated 400 ml of medium in each reactor with 1 ml early exponential phase cultures that were previously diluted 1∶100 from six overnight cultures of individual clones. The reactors were stirred at 800 rpm, aerated with sterile air, and the temperature was controlled to be at 37°C. Growth as batch cultures was allowed for 3.5 h. Then fresh medium was pumped into the reactors at 104 ml/h (0.26 volume changes per hour, corresponding to a doubling time of 2.66 h), and total volume in the reactors was kept constant at 400 ml. After 16 h, pumping speed was changed for three of the six reactors to be 384 ml/h (0.96 volume changes per hour, corresponding to a doubling time of 0.72 h). After 6 h, 0.05 µg/ml ciprofloxacin was added to the medium supply, and all reactors were spiked with ciprofloxacin to a final concentration of 0.05 µg/ml, to keep the amount of the antibiotic constant in all replicates. However, we cannot rule out the possibility that metabolic differences between the slow and fast growing chemostat populations could lead to differences in the pharmacokinetics of the antibiotic. Samples were taken every 30 min, optical density at 600 nm was determined, samples were diluted up to 1∶10^7^ in a series of 1∶10 dilutions, and 5 µl of each dilution were spotted on LB plates. After the spots dried, plates were incubated at 37°C overnight. Spots with numbers of colonies suitable for counting were then identified, and the number of colony forming units for every time point was calculated and normalized to the total number of cells as determined by the measured OD.

### Flow Cytometry

For the experiment in Figure S10, overnight cultures were diluted 1∶20 in LB Lennox, and analyzed in a flow cytometer (LSRII, Becton Dickinson) at an OD 600 nm of 0.9. Bacteria were identified by side scatter, and GFP emission was measured at 530 nm. Data were analyzed using FlowJo software (Tree Star, Inc.).

## Supporting Information

Figure S1 Schematic drawing of the microfluidic device used in this study. (A), (B), and (C) show a schematic drawing of the microfluidic device used at three different degrees of magnification. Bacterial cells grow in narrow dead-end channels (approximately 1 µm in width and height, 25 µm in length) that open on one side into a main trench (approximately 100 µm in width, 20 µm in height, drawing not to scale). Medium is flowing through the main trench at 2 ml/h (as illustrated by grey arrows in the drawings). This design allows the observation of the bottom cell in the channel for the whole duration of an experiment, whereas its progeny will eventually be pushed into the main trench and flushed away.
(TIF)

Figure S2 No antibiotic tolerance is observed in a genetically avirulent mutant. Quantitative analysis of an experiment analogous to the experiment shown in Figure 1, but using the genetically avirulent *ΔhilD* mutant. No induction of *ttss-1* is observed during growth in spent LB, and no cells survive exposure to 0.05 µg/ml ciprofloxacin. Blue, yellow, and red lines at the top of the plot indicate growth in LB, spent LB, and spent LB+0.05 µg/ml ciprofloxacin, respectively. Color-coding is scaled to GFP intensities from the experiment in Figure 1. *N* = 108.
(TIF)

Figure S3 Survival of antibiotic exposure is unlikely to be caused by resistance mutations. Experiment analogous to the one shown in Figure 1, except that cells surviving the first exposure to 0.05 µg/ml ciprofloxacin were then exposed to the same drug a second time. Cells were first grown in LB (blue segment at the top of the plot), medium was then changed to spent LB (yellow segment), followed by spent LB+0.05 µg/ml ciprofloxacin (first red segment). Again, survival is positively correlated with *ttss-1* expression (logistic regression with ANOVA, *p* = 3.0×10^−12^, *N* = 103) and negatively correlated with single cell elongation rates (logistic regression with ANOVA, *p* = 9.2×10^−3^, *N* = 106). Surviving cells were then allowed to regrow in LB (second blue segment) and subsequently challenged with LB+0.05 µg/ml ciprofloxacin (second red segment), followed by LB (third blue segment). None of the cells surviving the first exposure resumed division after the second exposure, showing that survival is most probably not caused by genetic resistance.
(TIF)

Figure S4 Survival of antibiotic exposure is not specific to one antibiotic class. Experiment analogous to the one shown in Figure 1, except that cells were subjected to kanamycin instead of ciprofloxacin. Blue, yellow, and red lines at the top of the plot indicate growth in LB, spent LB, and spent LB+16 µg/ml kanamycin, respectively. Survival was again positively correlated with expression of *ttss-1* as measured by GFP intensity (logistic regression with ANOVA, *p* = 3.7×10^−12^, *N* = 127), and negatively correlated with single cell elongation rates (logistic regression with ANOVA, *p* = 6.7×10^−7^, *N* = 128).
(TIF)

Figure S5 Antibiotic dose responses of wild type and *ΔhilD*. Dose responses for wild type were assayed in ciprofloxacin and kanamycin (A and B), and for *ΔhilD* in ciprofloxacin (C), using the procedure described in Text S1. Each plot indicates estimated 99% confidence intervals of IC_95_—that is, the dosage at which growth is inhibited by 95% as compared to drug-free growth, as a red bar. Upper and lower regression envelopes for α = 0.01 are indicated using grey regions. An asterisk in each figure indicates the lowest dosage used in the main test for the respective drug. We defined MIC to be the smallest dosage datum (the lowest of the *d_i_* values, see Text S1) above the estimated IC_95_ and chose twice this value for the lowest drug concentrations used.
(TIF)

Figure S6 Tolerance of T1^+^ cells is also observed at a clinically relevant kanamycin concentration. Results of an experiment analogous to the one shown in Figure S4, except that cells were exposed to a higher kanamycin concentration, 50 µg/ml. *ttss-1* expression levels were determined in 1,533 cells (measured as GFP fluorescence intensity at the last time point during antibiotic exposure), and their fate after exposure to antibiotics was observed. The histogram shows the number of cells in different GFP intensity categories, indicating *ttss-1* expression levels. Background fluorescence intensity (measured in areas of the image that do not contain cells) was subtracted from measured GFP intensity values. Color-coding denotes the probabilities to survive exposure to 50 µg/ml kanamycin for each GFP intensity category. Cells that express *ttss-1* have a significantly higher survival probability (logistic regression with ANOVA, *p*<2.2×10^−16^; *N* = 1,533). In addition, columns were assigned visually to two categories according to their GFP expression (“GFP on,” “GFP off”), and the percentage of cells surviving in the different categories was calculated.
(TIF)

Figure S7 Growth rate difference between subpopulations explains antibiotic tolerance. *ΔhilD* cells were grown in chemostats at two different growth rates, corresponding to those measured for T1^+^ (“slow,” filled circles, three independent replicates) and T1^−^ (“fast,” filled squares, three independent replicates). Growth rates in the chemostats were 0.96 h^−1^ and 0.26 h^−1^ for “fast” and “slow,” respectively; see Materials and Methods for how doubling times of the two subpopulations were determined. We added 0.05 µg/ml ciprofloxacin at time 0, and the number of colony forming units (cfu) was assessed by plating samples from different time points.
(TIF)

Figure S8 Growth retardation by gratuitous protein expression can also lead to antibiotic tolerance. *ΔhilD* cells carrying the a plasmid encoding LacZ under control of the lac promoter were subjected to the same experimental conditions as in Figure 1, except that different concentrations of IPTG were added to the spent LB. Higher concentrations of IPTG lead to stronger expression of *lacZ*, which in turn leads to growth retardation. Cell elongation rate is negatively correlated with survival after antibiotic exposure (logistic regression with ANOVA, *p*<2.2×10^−16^, *N* = 329). The black curve shows the survival probability conditional on the elongation rate. The histograms show the number of cells surviving or dying, respectively, in the different categories for cell elongation rate. Color-coding of the histogram indicates different IPTG concentrations.
(TIF)

Figure S9 Numbers of viable cells in the competition experiment. Numbers of viable cells for the experiment shown in Figure 3, as calculated by the number of colonies on LB plates and the respective dilution factors. After 3.5 h of growth in LB, all cultures were diluted 1∶100 in spent LB (blue boxes) and in spent LB containing 0.05 µg/ml ciprofloxacin (red boxes). Boxplots as in Figure 3; 20 independent replicates.
(TIF)

Figure S10 Cells carrying a kanamycin resistance marker in the *lpfED* locus show the same SPI-1 expression pattern as wild type. (A) Flow cytometry plots for representative samples of wild-type, *ΔlpfED*, and *ΔhilD* cells carrying the plasmid *psicA gfp*. Wild type and *ΔlpfED* showed indistinguishable expression patterns. (B) Quantitation of three independent replicate flow cytometric measurements of the strains used in (A) (*ΔhilD* is not shown, as its fraction of T1^+^ cells is per definition 0%). Gating was performed on a histogram obtained by analyzing *ΔhilD* cells; every count exceeding the distribution measured there was scored as a T1^+^ individual. Strains were diluted from overnight cultures in fresh LB Lennox and assayed at an optical density (600 nm) of 0.9.
(TIF)

Table S1 List of strains used in this study.
(DOCX)

Movie S1 Growth and survival of *S. typhimurium* cells exposed to 0.05 µg/ml ciprofloxacin. Bacteria are grown in the conditions indicated in the top left corner (LB – spent LB – spent LB+0.05 µg/ml ciprofloxacin – LB). Time between frames is 5 min. The timer in the top left corner indicates hh:mm:ss.
(AVI)

Movie S2 Survival of ciprofloxacin exposure is not caused by resistance mutations. Bacteria are grown in the conditions indicated in the top left corner (LB – spent LB – spent LB+0.05 µg/ml ciprofloxacin – LB – LB+0.05 µg/ml ciprofloxacin). Time between frames is 5 min. The timer in the top left corner indicates hh:mm:ss.
(AVI)

Movie S3 Growth and survival of *S. typhimurium* cells exposed to 16 µg/ml kanamycin. Bacteria are grown in the conditions indicated in the top left corner (LB – spent LB – spent LB+16 µg/ml kanamycin – LB). Time between frames is 5 min. The timer in the top left corner indicates hh:mm:ss.
(AVI)

Movie S4 Growth and survival of *S. typhimurium* cells exposed to 10 µg/ml ciprofloxacin. Bacteria are grown in the conditions indicated in the top left corner (LB – spent LB – spent LB+10 µg/ml ciprofloxacin – LB). Time between frames is 5 min. The timer in the top left corner indicates hh:mm:ss.
(AVI)

Movie S5 Growth and survival of *S. typhimurium* expressing gratuitous protein when exposed to 0.05 µg/ml ciprofloxacin. Bacteria are grown in the conditions indicated in the top left corner (LB – spent LB+1 mM ITPG – spent LB+1 mM IPTG+0.05 µg/ml ciprofloxacin – LB). Time between frames is 5 min. The timer in the top left corner indicates hh:mm:ss.
(AVI)

Text S1 Statistical analysis of antibiotic dose responses.
(PDF)

## Abbreviations

GFP: green fluorescent protein
MIC: minimal inhibitory concentration
*S. typhimurium*: *Salmonella enterica* subspecies I serovar Typhimurium
T1^+^ cells: cells expressing *ttss-1*
T1^−^ cells: cells not expressing *ttss-1*
*ttss-1*: type three secretion system 1
