# Supplementary figures and images for: Bistable Expression of Virulence Genes in Salmonella Leads to the Formation of an Antibiotic-Tolerant Subpopulation
Source: PLoS Biol. 2014 Aug 19;12(8):e1001928. doi: 10.1371/journal.pbio.1001928 (PMC4138020; doi:10.1371/journal.pbio.1001928)

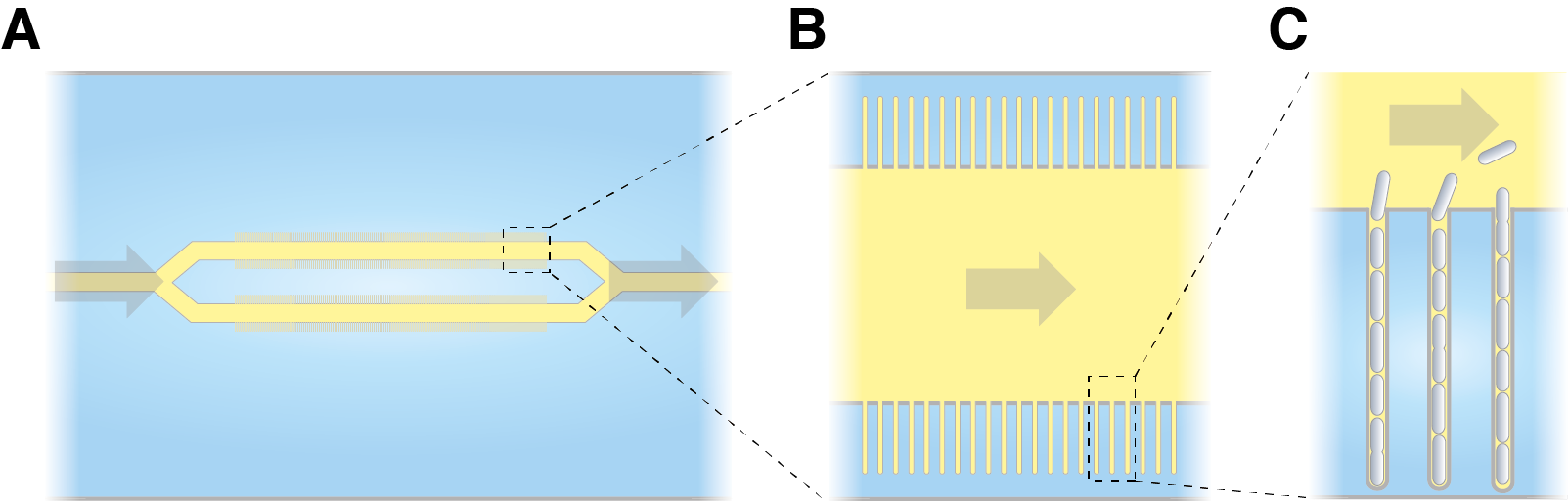

Supplement: Figure S1 — Schematic drawing of the microfluidic device used in this study. (A), (B), and (C) show a schematic drawing of the microfluidic device used at three different degrees of magnification. Bacterial cells grow in narrow dead-end channels (approximately 1 µm in width and height, 25 µm in length) that open on one side into a main trench (approximately 100 µm in width, 20 µm in height, drawing not to scale). Medium is flowing through the main trench at 2 ml/h (as illustrated by grey arrows in the drawings). This design allows the observation of the bottom cell in the channel for the whole duration of an experiment, whereas its progeny will eventually be pushed into the main trench and flushed away. (TIF) [file pbio.1001928.s001.tif]

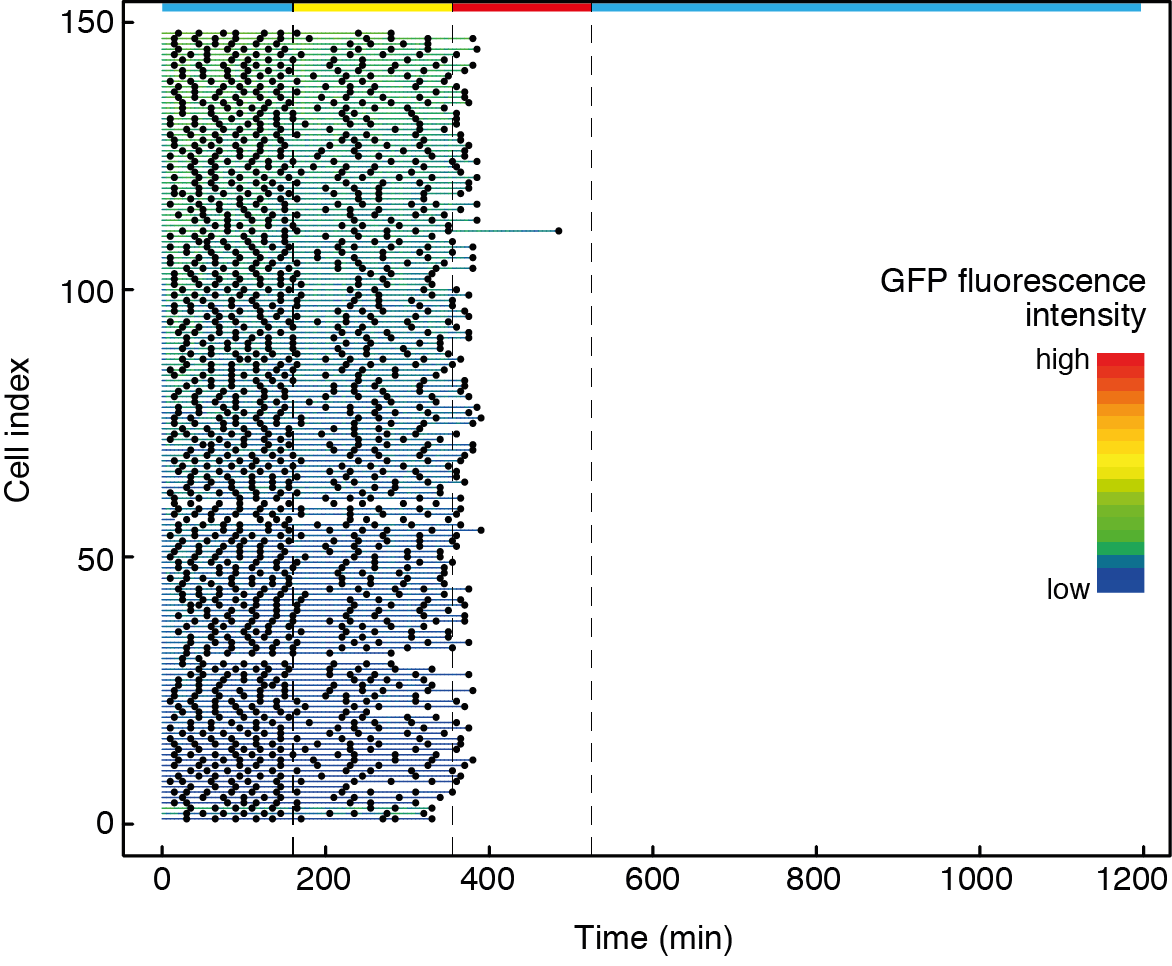

Supplement: Figure S2 — No antibiotic tolerance is observed in a genetically avirulent mutant. Quantitative analysis of an experiment analogous to the experiment shown in Figure 1, but using the genetically avirulent ΔhilD mutant. No induction of ttss-1 is observed during growth in spent LB, and no cells survive exposure to 0.05 µg/ml ciprofloxacin. Blue, yellow, and red lines at the top of the plot indicate growth in LB, spent LB, and spent LB+0.05 µg/ml ciprofloxacin, respectively. Color-coding is scaled to GFP intensities from the experiment in Figure 1. N = 108. (TIF) [file pbio.1001928.s002.tif]

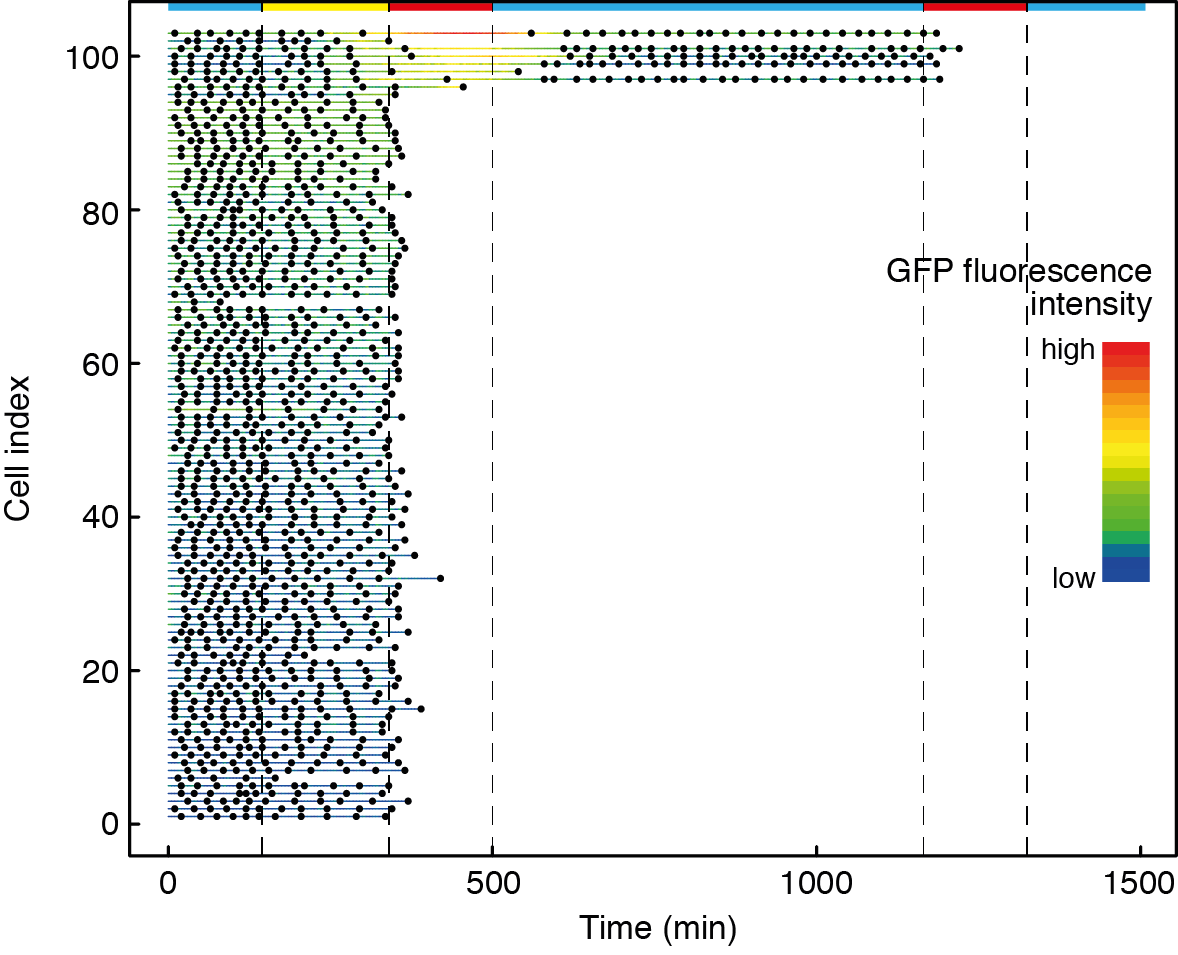

Supplement: Figure S3 — Survival of antibiotic exposure is unlikely to be caused by resistance mutations. Experiment analogous to the one shown in Figure 1, except that cells surviving the first exposure to 0.05 µg/ml ciprofloxacin were then exposed to the same drug a second time. Cells were first grown in LB (blue segment at the top of the plot), medium was then changed to spent LB (yellow segment), followed by spent LB+0.05 µg/ml ciprofloxacin (first red segment). Again, survival is positively correlated with ttss-1 expression (logistic regression with ANOVA, p = 3.0×10^−12^, N = 103) and negatively correlated with single cell elongation rates (logistic regression with ANOVA, p = 9.2×10^−3^, N = 106). Surviving cells were then allowed to regrow in LB (second blue segment) and subsequently challenged with LB+0.05 µg/ml ciprofloxacin (second red segment), followed by LB (third blue segment). None of the cells surviving the first exposure resumed division after the second exposure, showing that survival is most probably not caused by genetic resistance. (TIF) [file pbio.1001928.s003.tif]

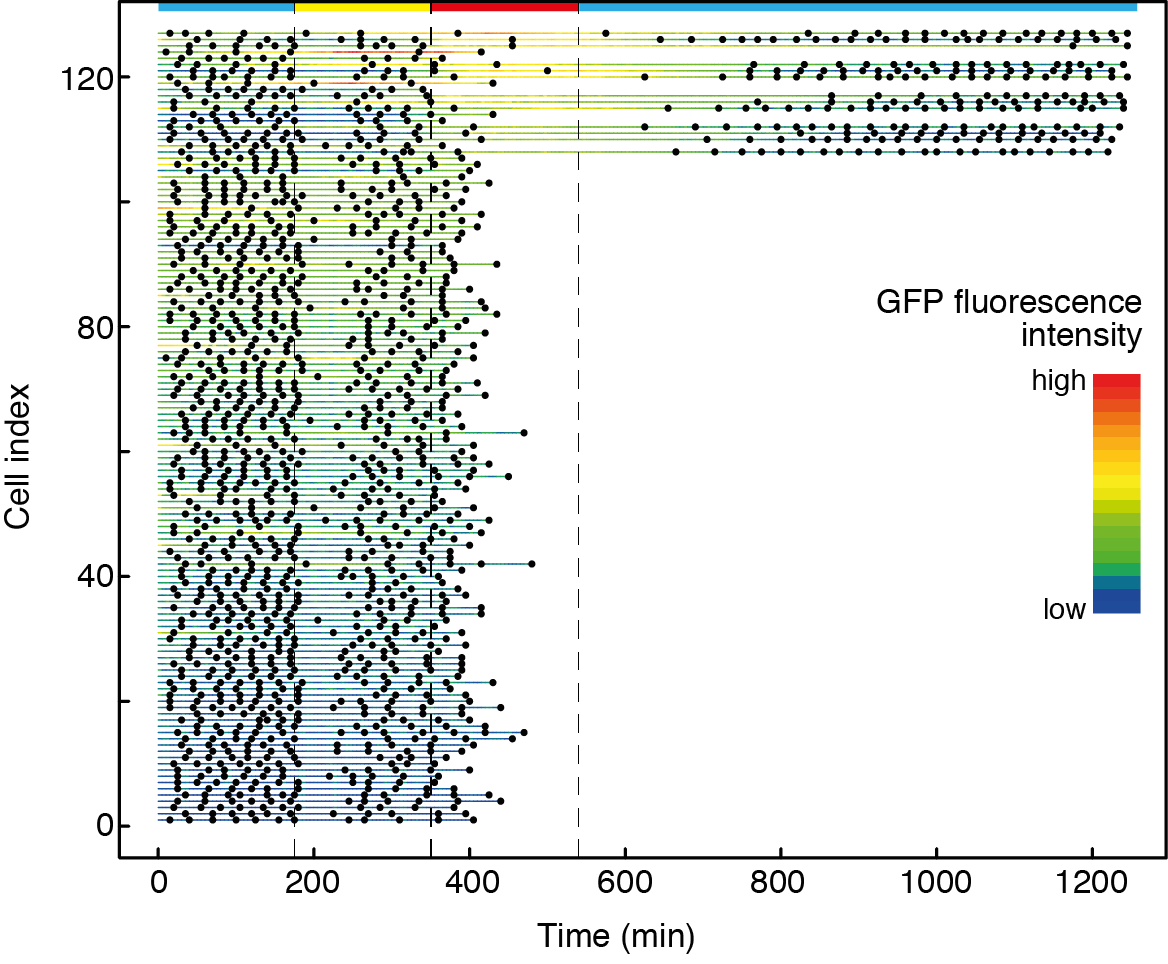

Supplement: Figure S4 — Survival of antibiotic exposure is not specific to one antibiotic class. Experiment analogous to the one shown in Figure 1, except that cells were subjected to kanamycin instead of ciprofloxacin. Blue, yellow, and red lines at the top of the plot indicate growth in LB, spent LB, and spent LB+16 µg/ml kanamycin, respectively. Survival was again positively correlated with expression of ttss-1 as measured by GFP intensity (logistic regression with ANOVA, p = 3.7×10^−12^, N = 127), and negatively correlated with single cell elongation rates (logistic regression with ANOVA, p = 6.7×10^−7^, N = 128). (TIF) [file pbio.1001928.s004.tif]

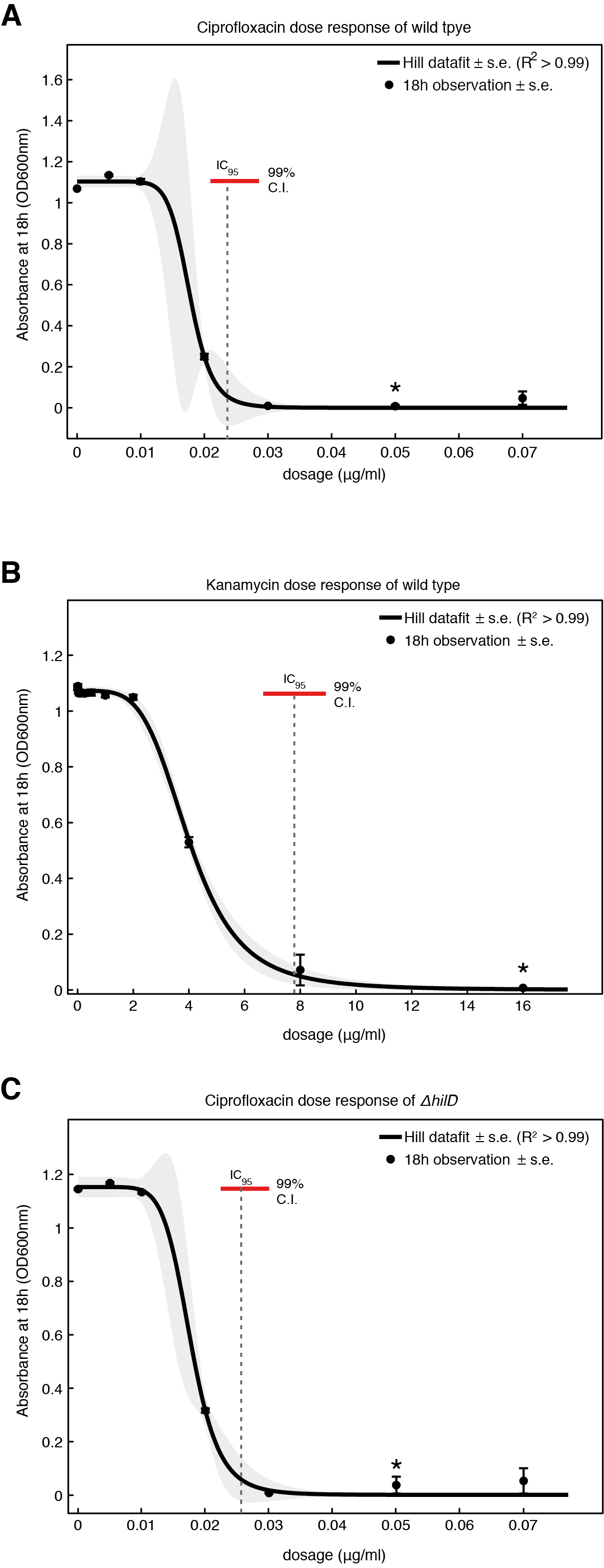

Supplement: Figure S5 — Antibiotic dose responses of wild type and ΔhilD. Dose responses for wild type were assayed in ciprofloxacin and kanamycin (A and B), and for ΔhilD in ciprofloxacin (C), using the procedure described in Text S1. Each plot indicates estimated 99% confidence intervals of IC95—that is, the dosage at which growth is inhibited by 95% as compared to drug-free growth, as a red bar. Upper and lower regression envelopes for α = 0.01 are indicated using grey regions. An asterisk in each figure indicates the lowest dosage used in the main test for the respective drug. We defined MIC to be the smallest dosage datum (the lowest of the di values, see Text S1) above the estimated IC95 and chose twice this value for the lowest drug concentrations used. (TIF) [file pbio.1001928.s005.tif]

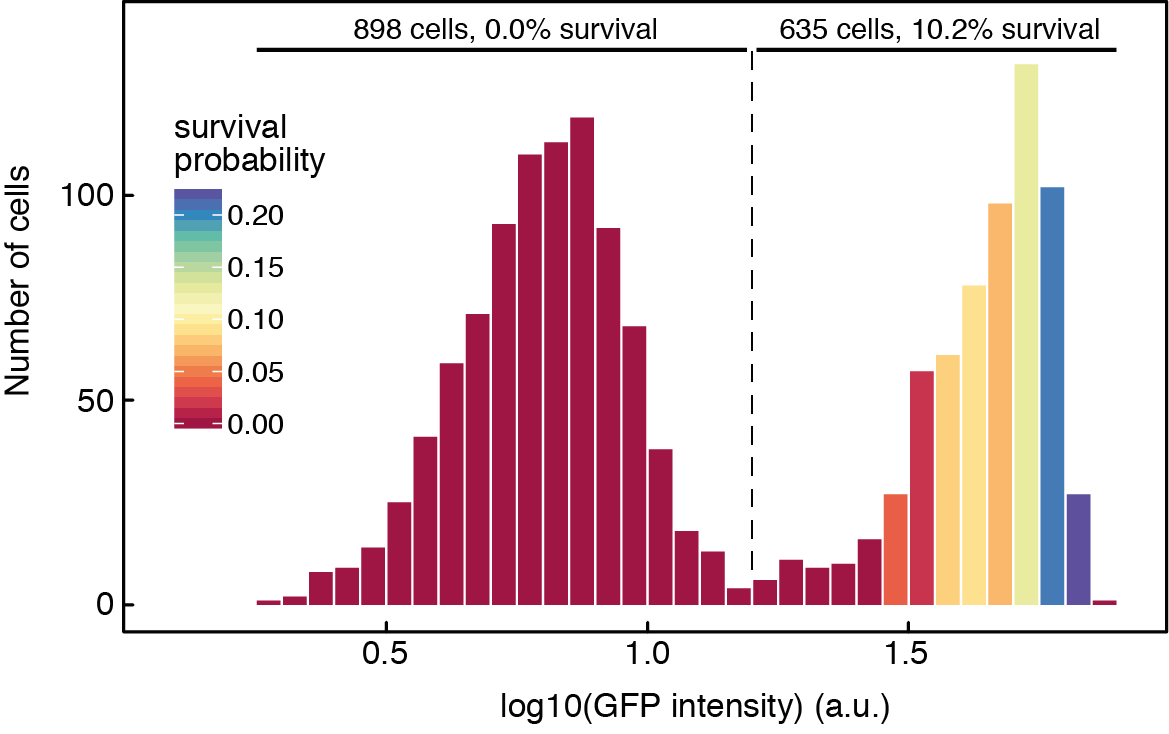

Supplement: Figure S6 — Tolerance of T1+ cells is also observed at a clinically relevant kanamycin concentration. Results of an experiment analogous to the one shown in Figure S4, except that cells were exposed to a higher kanamycin concentration, 50 µg/ml. ttss-1 expression levels were determined in 1,533 cells (measured as GFP fluorescence intensity at the last time point during antibiotic exposure), and their fate after exposure to antibiotics was observed. The histogram shows the number of cells in different GFP intensity categories, indicating ttss-1 expression levels. Background fluorescence intensity (measured in areas of the image that do not contain cells) was subtracted from measured GFP intensity values. Color-coding denotes the probabilities to survive exposure to 50 µg/ml kanamycin for each GFP intensity category. Cells that express ttss-1 have a significantly higher survival probability (logistic regression with ANOVA, p<2.2×10^−16^; N = 1,533). In addition, columns were assigned visually to two categories according to their GFP expression (“GFP on,” “GFP off”), and the percentage of cells surviving in the different categories was calculated. (TIF) [file pbio.1001928.s006.tif]

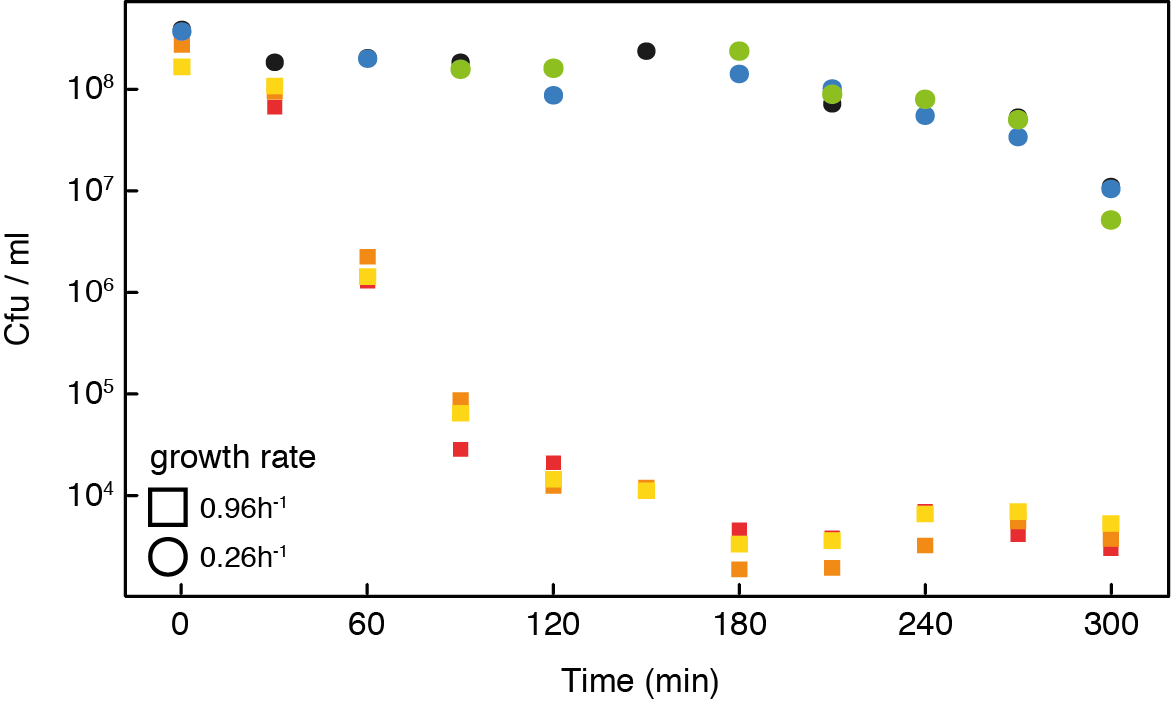

Supplement: Figure S7 — Growth rate difference between subpopulations explains antibiotic tolerance. ΔhilD cells were grown in chemostats at two different growth rates, corresponding to those measured for T1+ (“slow,” filled circles, three independent replicates) and T1− (“fast,” filled squares, three independent replicates). Growth rates in the chemostats were 0.96 h−1 and 0.26 h−1 for “fast” and “slow,” respectively; see Materials and Methods for how doubling times of the two subpopulations were determined. We added 0.05 µg/ml ciprofloxacin at time 0, and the number of colony forming units (cfu) was assessed by plating samples from different time points. (TIF) [file pbio.1001928.s007.tif]

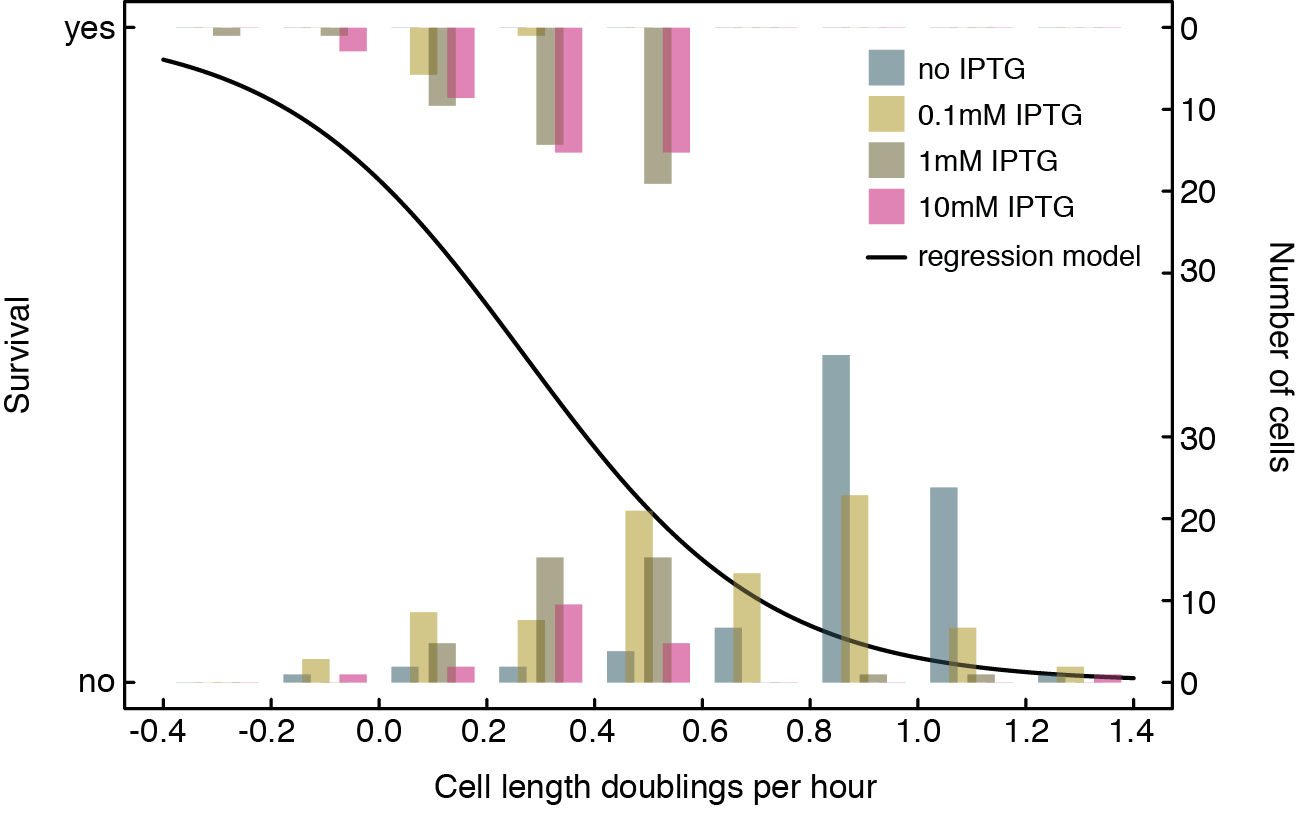

Supplement: Figure S8 — Growth retardation by gratuitous protein expression can also lead to antibiotic tolerance. ΔhilD cells carrying the a plasmid encoding LacZ under control of the lac promoter were subjected to the same experimental conditions as in Figure 1, except that different concentrations of IPTG were added to the spent LB. Higher concentrations of IPTG lead to stronger expression of lacZ, which in turn leads to growth retardation. Cell elongation rate is negatively correlated with survival after antibiotic exposure (logistic regression with ANOVA, p<2.2×10^−16^, N = 329). The black curve shows the survival probability conditional on the elongation rate. The histograms show the number of cells surviving or dying, respectively, in the different categories for cell elongation rate. Color-coding of the histogram indicates different IPTG concentrations. (TIF) [file pbio.1001928.s008.tif]

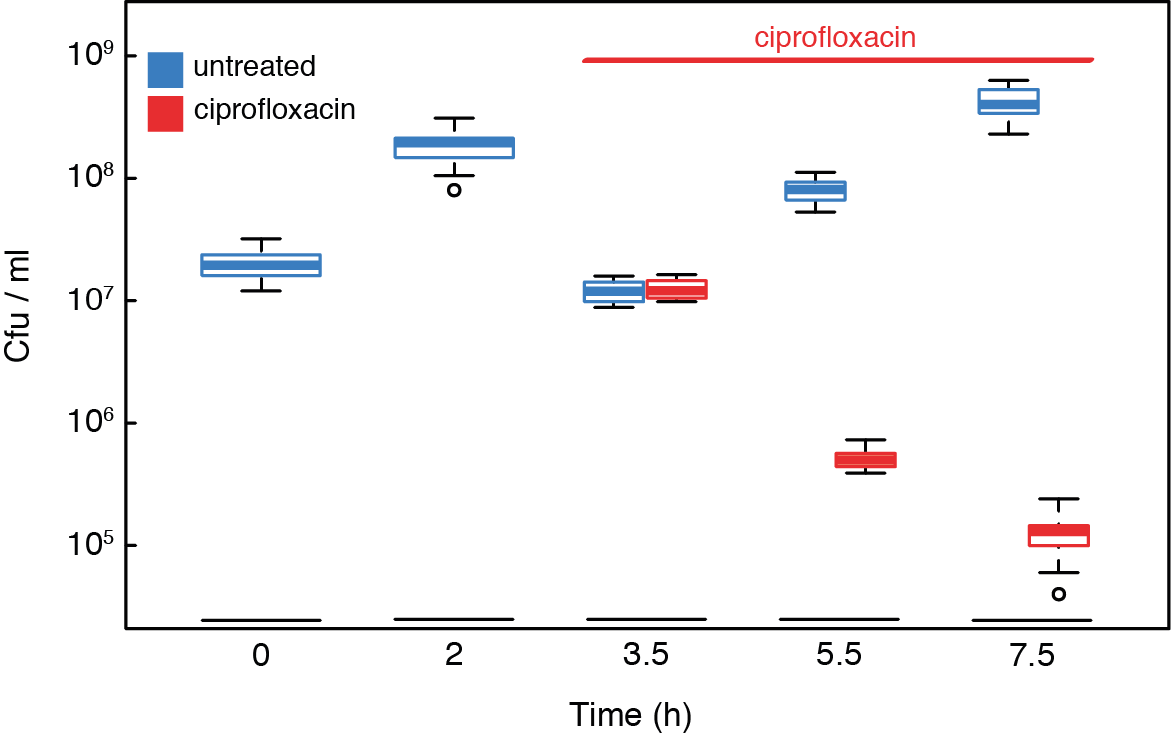

Supplement: Figure S9 — Numbers of viable cells in the competition experiment. Numbers of viable cells for the experiment shown in Figure 3, as calculated by the number of colonies on LB plates and the respective dilution factors. After 3.5 h of growth in LB, all cultures were diluted 1∶100 in spent LB (blue boxes) and in spent LB containing 0.05 µg/ml ciprofloxacin (red boxes). Boxplots as in Figure 3; 20 independent replicates. (TIF) [file pbio.1001928.s009.tif]

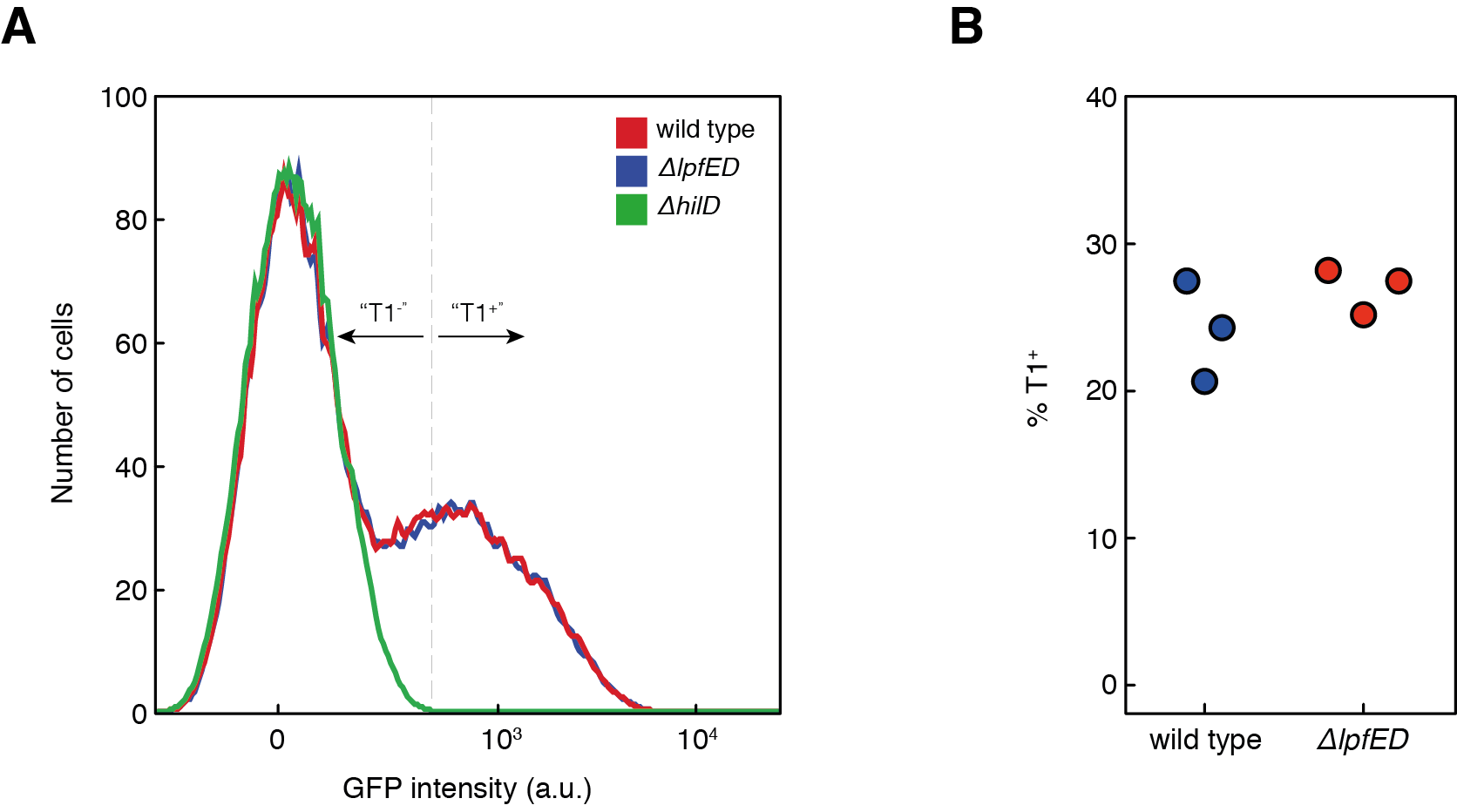

Supplement: Figure S10 — Cells carrying a kanamycin resistance marker in the lpfED locus show the same SPI-1 expression pattern as wild type. (A) Flow cytometry plots for representative samples of wild-type, ΔlpfED, and ΔhilD cells carrying the plasmid psicA gfp. Wild type and ΔlpfED showed indistinguishable expression patterns. (B) Quantitation of three independent replicate flow cytometric measurements of the strains used in (A) (ΔhilD is not shown, as its fraction of T1+ cells is per definition 0%). Gating was performed on a histogram obtained by analyzing ΔhilD cells; every count exceeding the distribution measured there was scored as a T1+ individual. Strains were diluted from overnight cultures in fresh LB Lennox and assayed at an optical density (600 nm) of 0.9. (TIF) [file pbio.1001928.s010.tif]
