# Supplementary material for: Bistable Expression of Virulence Genes in Salmonella Leads to the Formation of an Antibiotic-Tolerant Subpopulation
Source: PLoS Biol. 2014 Aug 19;12(8):e1001928. doi: 10.1371/journal.pbio.1001928 (PMC4138020; doi:10.1371/journal.pbio.1001928)
Supplement: Table S1 — List of strains used in this study. (DOCX) [file pbio.1001928.s011.docx]

| **Strain name** | **Genotype** | **Reference** |
| --- | --- | --- |
| SB300 (SL1344) | *rpsL hisG* | Hoiseth and Stocker, Nature 1981 [1] |
| M2007 | SB300 *hilD::aphT* | Sturm et al., PLoS Pathog, 2007 [2] |
| M3067 | SB300 *lpfED::aphT* | this work |
| Z19 | SB300 *ΔhilD* | this work |
| X6802 | *rpsL hisG ΔfliC825 ΔfljB217* | Curtiss and Kang, US Pat. No. 7195757, 2007 [3] |
| M3139 | X8602 *ΔhilD* | this work |

**Table S1. List of strains used in this study.**

1. Hoiseth SK, Stocker BAD (1981) Aromatic-dependent Salmonella typhimurium are non-virulent and effective as live vaccines. Nature 291: 238–239.

2. Sturm A, Heinemann M, Arnoldini M, Benecke A, Ackermann M, et al. (2011) The Cost of Virulence: Retarded Growth of Salmonella Typhimurium Cells Expressing Type III Secretion System 1. PLoS Pathog 7: e1002143. doi:10.1371/journal.ppat.1002143.

3. Curtiss R III, Kang HY (2007) Modulation of immune responses to foreign antigens expressed by recombinant attenuated bacterial vectors. US Patent No 7195757 B2.
