# Supplementary material for: Bistable Expression of Virulence Genes in Salmonella Leads to the Formation of an Antibiotic-Tolerant Subpopulation
Source: PLoS Biol. 2014 Aug 19;12(8):e1001928. doi: 10.1371/journal.pbio.1001928 (PMC4138020; doi:10.1371/journal.pbio.1001928)
Supplement: Text S1 — Statistical analysis of antibiotic dose responses. (PDF) [file pbio.1001928.s017.pdf]

QUANTIFYING VARIATION IN IC$_x$ VALUES

A dose-response dataset is a collection of dosages d_i for $i = 0, ..., N$ that satisfy $0 = d_0 < d_1 < ... < d_N$. Population density data at some defined time T, taken here to be $18h$, is held in an $N \times M$ matrix $\mathcal{D} := (D_{ij})$, representing data taken from a microtitre plate-reading device. These are determined empirically for $j = 1, ..., M$, M being the number of replicates of each bacterial culture at each dose. An inhibition coefficient, or IC_x, is a value of the dose that reduces population density by $x\%$ relative to drug-free growth. Other growth measures could be used, for example, an estimate of exponential growth rate.

To approximate IC_x the following is done. First, the mean density of the zero-drug control is determined, $\overline{D_0} := \frac{1}{M}\sum_{j=1}^{M} D_{0j}$, one then chooses a putative model $F(\cdot)$ of the data. This must be a function such that the approximation $D_{ij} \approx F(d_j)$ is possible with respect to some metric (and below we require that the regression coefficient $R^2 > 0.99$ for this). A standard choice for F is a Hill function, whereby

$$F(d) = \Delta \frac{K^n}{d^n + K^n}.$$

This has many of the properties desired by a dose-response in the sense that it is a monotone decreasing function that satisfies $F(d) > 0, F(0) = \Delta > 0$ and $\lim_{d\to\infty} F(d) = 0$. Other choices are possible, but a Hill function approach is common. Then, given that x is expressed as a percentage, solve for d in $F(d) = (x\overline{D_0})/100$, it follows that $IC_x \approx d$. In other words,

$$F(IC_x) = (x\overline{D_0})/100.$$

When this solution exists, it is unique because F is a decreasing function.

Given α, using nonlinear regression we then estimate $100\cdot(1-\alpha)\%$-confidence intervals that estimate upper and lower envelopes, $F^-(d)$ and $F^+(d)$ of the predicted dose-response at each dose. In general, these are non-monotone functions that satisfy $F^-(d) < F(d) < F^+(d)$. An estimate of the confidence interval of IC_x is then given by the interval (d_-, d_+) where

$$(F^-)^{-1}(d_-) = (x\overline{D_0})/100 = (F^+)^{-1}(d_+).$$

This procedure was implemented in Matlab using the `NonLinearModel` class from the Statistics Toolbox, and the results are presented in Figure S5.
